# Management of Functional Neurological Disorders: Turkiye Experience

**DOI:** 10.1192/j.eurpsy.2025.1260

**Published:** 2025-08-26

**Authors:** I. Yildiz, G. Yalçin Çakmakli, A. Fil Balkan, T. Demirsöz, B. Elibol, M. K. Yazici

**Affiliations:** 1 psychiatry; 2 neurology; 3 physical therapy and rehabilitation, hacettepe university, Ankara, Türkiye

## Abstract

**Introduction:**

Functional neurological disorder (FND) is diagnosed in approximately one-third of admissions to the general neurology outpatient clinic. Although it affects both gender in a wide age range, it occurs more frequently in women between ages of 35 and 50. As a common neuropsychiatric disorder affecting mostly young people at productive ages, FND also leads to an important socioeconomic burden. For this reason, clinical and research activities tended to increase recently in order to develop new neuroscientific approaches for the treatment of this disorder. Various new clinical centers that offer specialized and multidisciplinary treatment to FND patients, have been established around the world. Likewise, a study group was established in Hacettepe University Adult Hospital, where FND patients were started to be treated by a multidisciplinary team, by also considering the specific needs of our country’s population.

**Objectives:**

Here, we aim to share the phenomenological and clinical characteristics and treatment response of patients with functional neurological disorders (FND) who received short-term inpatient multidisciplinary treatment in a university hospital between 2020 and 2023.

**Methods:**

FND study group at Hacettepe University Adult Hospital was founded by 2 neurologists, 2 psychiatrists, 1 psychologist and 2 physiotherapists.

Within the framework of the protocol, FND patients were first informed about the disease process by a neurologist and a psychiatrist and then followed up for further need for an inpatient multidisciplinary approach.

During hospital admission in the room reserved for FND patients in the neurology ward, the treatment protocol was implemented in such a way that each session included **one hour of cognitive behavioral therapy-based psychotherapy** and **at least 2 hours of intensive physiotherapy including FND-specific approaches** for at least 5 days.

Demographic and clinical characteristics of the patients were noted, their fitness levels before and after treatment were evaluated with a visual analogue scale, and the patients’ perceived improvement levels were also evaluated with VAS and recorded as a percentage.

**Results:**

The majority of patients were women (19/11).

The age range was wide, between 19 to 75 (average 44 years).

Patients were classified under four groups based on the predominant symptom (gait disorders:15, fixed dystonia: 9, swallowing and speech problems:3, sensorial problems:3)

The level of improvement perceived by the patients was the best in those with sensorial problems, and the worst in those with swallowing and speech problems (%40).

Those with the lowest level of perceived wellness in terms of general health were patients with dystonia component.

**Conclusions:**

Multidisciplinary FND rehabilitation may provide different degrees of benefit to FND patients of different subtypes. Our results may have the potential to contribute to clinicians and academicians in predicting treatment outcomes.

**Disclosure of Interest:**

None Declared

